# PV Panel Model Parameter Estimation by Using Particle Swarm Optimization and Artificial Neural Network

**DOI:** 10.3390/s24103006

**Published:** 2024-05-09

**Authors:** Wai-Lun Lo, Henry Shu-Hung Chung, Richard Tai-Chiu Hsung, Hong Fu, Tak-Wai Shen

**Affiliations:** 1 Department of Computer Science, Hong Kong Chu Hai College, Hong Kong, China; richardhsung@chuhai.edu.hk (R.T.-C.H.); twshen@chuhai.edu.hk (T.-W.S.); 2 Department of Electrical Engineering, City University of Hong Kong, Hong Kong, China; eeshc@cityu.edu.hk; 3 Department of Mathematics and Information Technology, The Education University of Hong Kong, Hong Kong, China; hfu@eduhk.hk

**Keywords:** model parameters estimation, neural network, particle swarm optimization, photovoltaic panel

## Abstract

Photovoltaic (PV) panels are one of the popular green energy resources and PV panel parameter estimations are one of the popular research topics in PV panel technology. The PV panel parameters could be used for PV panel health monitoring and fault diagnosis. Recently, a PV panel parameters estimation method based in neural network and numerical current predictor methods has been developed. However, in order to further improve the estimation accuracies, a new approach of PV panel parameter estimation is proposed in this paper. The output current and voltage dynamic responses of a PV panel are measured, and the time series of the I–V vectors will be used as input to an artificial neural network (ANN)-based PV model parameter range classifier (MPRC). The MPRC is trained using an I–V dataset with large variations in PV model parameters. The results of MPRC are used to preset the initial particles’ population for a particle swarm optimization (PSO) algorithm. The PSO algorithm is used to estimate the PV panel parameters and the results could be used for PV panel health monitoring and the derivation of maximum power point tracking (MMPT). Simulations results based on an experimental I–V dataset and an I–V dataset generated by simulation show that the proposed algorithms can achieve up to 3.5% accuracy and the speed of convergence was significantly improved as compared to a purely PSO approach.

## 1. Introduction

Solar energy is one of the cleanest energy solutions in renewable energy sources. The photovoltaic (PV) system is widely applied in industrial and commercial sectors nowadays due to the rapid advancements in PV cell technology, energy conversion efficiency and power electronics control methods for maximum power point tracking (MPPT). PV panel models’ parameters estimations, MMPT control and fault diagnosis are among the most popular research topics in renewable energy systems. There has been past research in applying artificial neural networks (ANNs) in PV system modelling, parameter estimation and power output prediction. In [1], a new neural network-based solar cell modelling method is proposed. The temperature and irradiance are used as inputs for an ANN to estimate the PV panel circuit model parameters. An adaptive artificial neural network (ANN) for the modeling and simulation of a stand-alone photovoltaic (SAPV) system is proposed in [2] and the proposed method can be applied in variable climatic conditions for SAPV system sizing procedures. The application of a neural network to 24-h-ahead power generation forecasting for PV systems is investigated in [3]. In [4], the operating current of the PV panel is directly predicted from the general regression neural network (GRNN) model with the inputs of solar radiation, cell temperature and the operating voltage of the system. A radial basis function neural network (RBFNN) approach is proposed in [5] to estimate the output current and power based on the input data of radiation and voltage. In [6], based on a regularization performance function, the modified error is used in the Levenberg Marquardt optimization to update the weights and biases of the ANN, which uses the irradiance and temperature as inputs to predict the output power of a PV panel.

Laudani [7] proposed to estimate the PV panel circuit model parameters by using the parameters on the I–V curve of the PV panel. A comparison of the ANFIS and ANNs for parameters estimation of PV modules is given [8]. A new approach for the modelling and prediction of PV power output using an ANN with temperature and irradiance as inputs is proposed in [9]. In [10], the neural network uses the input parameters of hourly solar radiation intensity, the highest, the lowest and the average daily temperature, and hourly PV system power output to forecast the PV power output 24 h ahead. In [11], a new method for the deterministic and probabilistic forecasting of PV power based on a deep convolutional neural network was proposed. The research in [12] presents an ANN solution to predict the power generated by the photovoltaic system based on solar radiation measurements. In [13], the non-linear equations between PV model parameters and output voltage and current are formulated. The generalized Hopfield neural network (GHNN) optimization technique is used to estimate the five unknown PV model parameters. The energy function is used to extract the MPP at different environmental conditions by considering the varying nature of shunt resistance, series resistance and ideality factor.

In [14], a high-precision deep neural network model (PVPNet) is proposed for forecasting PV system output power and to generate a 24 h probabilistic and deterministic forecasting of PV power output based on meteorological information such as temperature, solar radiation and historical PV system output data. Based on the nonlinear least squares fitting algorithm, a new approach to estimate the optimal parameters of the photovoltaic (PV) modules using in-field outdoor measurements and manufacturers’ datasheet information is proposed in [15]. It can determine the optimal values for the model parameters of series resistance, reverse saturation current, photocurrent, ideality factor and shunt resistance. In [16], a study on the ANN modeling and analysis of the PV thermal system is given. A new algorithm [17] for PV panel model parameters estimation by using a neural network (ANN) with a numerical current prediction (NCP) layer is proposed in [17]. Output current and voltage signals (I–V) after load perturbation are observed and the ANN is trained to estimate the PV panel model parameters with accuracies of about 6%.

In order to ensure high energy conversion efficiencies for the PV panel systems, many MPPT control methods have been developed. The perturb and observe (P&O) method [18,19] and the incremental conductance method (IC) [20] are among the most popular approaches for the MPPT control of PV panel systems. There was also past research in modified P&O methods and ANN approaches in the adaptive MPPT control of PV panel systems. In [21], the use of system oscillation in a perturbation-based maximum power point (MPP) tracker is proposed for locating the MPP of photovoltaic (PV) panels. In [22], a new solar array modelling and MPPT tracking method using an ANN is proposed. The ANN used the temperature and solar irradiance as inputs to predict the MPPT voltage, current and power.

In [23], a new method for solar array modelling and MPPT control using an ANN is proposed. The ANN uses the temperature and solar irradiance to predict the maximum power, which acts as the setpoint for the PI control for the boost converter. Abouda [24] studies the design, simulation and voltage control of standalone PV system-based MPPT methods which can be applied to a pumping system.

The authors of [25] propose a modified P&O algorithm for the MPPT tracking of PV systems using a buck–boost converter under rapidly changing weather conditions. An adaptive voltage-sensor-based MPPT tracking algorithm employing a variable scaling factor for a single-ended primary-inductance converter (SEPIC) is proposed in [26]. An ANN method to predict the duty cycle for MPPT in PV systems is proposed in [27]. In [28], a new MPPT-based ANN for a photovoltaic system under partial shading condition is proposed. A scanning method is used to track the global maximum of a PV array system and the ANN is used to derive the duty cycle for the control of the DC/DC converter. A recurrent fuzzy cerebellar model articulation neural network (RFCMANN)-based controller to regulate the active and reactive power of a single-stage three-phase grid-connected photovoltaic (PV) system during grid faults is proposed in [29]. In [30], the speed of the induction motor is used as a feedback signal for a controller to generate the voltage, and the error and change in error obtained are used as inputs for the ANN to generate the switching signal to the SEPIC converter.

An ANN approach [31] has been proposed to predict the MPPT by considering different sets of ANN input parameters. The irradiance, electrical, thermal and weather parameters yielded a higher MPP identification accuracy. The research in [31] aims to develop a robust and practical PV MPPT identification tool by using reliable experimental datasets. The correlations between the voltage and the current at maximum power from one side, and the irradiance information, electrical parameters, thermal parameters and weather parameters from another side, are investigated and compared. Four combinations of various PV parameters using various ANN-based MPP identifiers are presented, and the accuracies of the MPP identification are compared by using the mean squared error (MSE). The ANN used the temperature and solar irradiance as inputs to predict the MPPT voltage, current and power. Two intelligent controllers based on an ANN and adaptive neuro-fuzzy inference system (ANFIS) are proposed to optimize the PV system’s output in non-uniform weather conditions and compared in [32].

ANN approaches have been developed for PV system fault detection. In [33], an ANN is used to predict the output voltage and current based on the temperature and solar irradiance. A PV panel is considered to be in a faulty state if the residual errors between the measured voltage and estimated voltage exceed threshold values. In [34], a method for the fault diagnosis approach for photovoltaic arrays based on unsupervised sample clustering and probabilistic neural network model is proposed. The Gaussian kernel function (GKF) has been introduced into the fuzzy C means (FCM) algorithm for a probabilistic neural network fault diagnosis model. The output of this method can be used for fault class identification. In [35], an intelligent real-time PV panel monitoring system using an ANN is proposed. An ANN with the temperature, solar irradiance and open circuit voltage as inputs is used to predict the output power. When the residual difference is above 10%, the system is considered to be in degradation. An automatic detection system for deteriorated PV modules that uses a drone with a thermal camera is proposed in [36]. In [37], one ANN stage is used to predict the single-diode-model parameters under the hypothesis of healthy operation and another one is used for the degraded condition. The variation in each parameter, of the outputs of the two ANN stages, will give a direct identification of the type of degradation in the PV panel.

Furthermore, past research has studied the application of the particle swarm optimization method (PSO) for PV panel parameter estimation and output prediction. A hybrid evolutionary optimization algorithm [38] has been presented for training an ANN to forecast the energy production of PV panels. In [39], the I–V curve, genetic algorithm is used to estimate the PV circuit model parameters so as to fit the estimated PV output quantities, such as short circuit current, open circuit voltage, maximum voltage, current and power, with the actual measured quantities. A method for identifying parameters for PV solar cells and modules using a flexible PSO algorithm is proposed in [40]. Dawan [41] compares the power output forecasting on a PV system using an adaptive neuro-fuzzy inference systems and a PSO–ANN model. 

PSO approaches have been developed for MPPT control and fault detection and diagnosis. In [42], an MPPT design using a PSO technique for PV system control is proposed. The proposed system is compared to the fuzzy logic and P&O controllers. In [43], an improved PSO-based MPPT strategy for PV systems is proposed. The initial positions of the particles for PSO are located by analyzing the relationship of the current vs. voltage and the power vs. voltage curves so that the initial particles are chosen to be near the MPPT. The proposed method can improve the speed for PSO convergence. The investigation in [44] proposes the tracking of the MPPT of the PV system under partial shading conditions through a novel heuristic optimization algorithm, the flower pollination algorithm. In [45], the optimal MPP voltage is estimated by the PSO and P&O perturbation methods. In [46], the P&O method is used to excite the PV system for each particle and the optimal voltage for maximum power is estimated by the PSO method. The PV panel parameters are estimated by applying the PSO on the voltage and current time series [47] and the PV parameter obtained could be used for fault diagnosis.

Most of the past research in PV model parameters estimation uses temperature and irradiance as inputs and PV model parameters or maximum power voltage as outputs for ANN. Some research for PV model parameters estimation by using the I–V dynamics as input data has been done. An ANN approach or modified PSO approach have been proposed. However, in this paper, a new PV panel model parameter estimation method using PSO and an ANN is proposed. The PSO algorithm used in the proposed system has a modified initialization stage in which the initial population is generated by using the outputs of a model parameter range classifier (MPRC). Firstly, the output current and voltage data after a load perturbation are measured; the current and voltage time series are then inputted to a model parameter range classifier (MPRC). The MPRC is an ANN which was trained by a large I–V dataset generated by load perturbation responses for different PV models’ parameters. The outputs of the MPRC will identify the parameter ranges of each of the model’s parameters. The initial population of the particles for PSO is selected based on the outputs of the MPRC. Simulation studies show that the accuracy of the proposed method is better than the purely ANN approach and the ANN–NCP method in [17]. Furthermore, the convergence speed of the proposed method is faster than that of the classical PSO approach. The estimation accuracies of the proposed method can achieve 4% with a smaller number of iterations as compared to the classical PSO approach. The major improvement of the PSO method is due to the initial model parameters’ pre-tuning by using the ANN, which could give near optimal solutions at the starting stage of the PSO algorithm. The organization of the paper is as follows. The proposed system structure and methodology are described in Section 2. The results and analysis are given in Section 3. The discussion is given in Section 4. The conclusion is given in Section 5.

## 2. Methodology

### 2.1. Modelling of PV Panel

**Figure 1:**
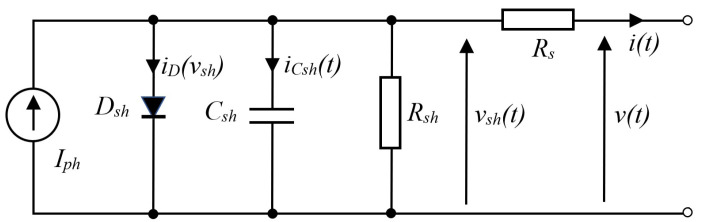
PV panel circuit model.

According to [17,47], a solar PV panel can be modelled by the electrical model in Figure 1 with modifications in parameters that consider the number of cells connected in series and/or parallel.

In Figure 1, *I_ph_* is the current source determined by incident light, *D_sh_*, *C_sh_* and *R_sh_* are the model p-n junction by diode, capacitor and resistor, and *R_s_* is the series resistance. *I_o_* is the reverse saturation current, *v_T_* = *n_id_ kT*/*q*, n_id_ is the ideality factor, *q* is the elementary charge, *k* is the Boltzmann constant, and *T* is the temperature of the p-n junction in Kelvin. Assume that the parasitic capacitance *C_g_* and resistance R_g_ between the frame of the panel and the terminals can be neglected (e.g., <1 nF and >10 MΩ). The circuit model equations are summarized as follows.

(1) $$i_{D}\left(v_{sh}\right)=I_{o}\left(e^{\frac{v_{sh}}{v_{T}}}-1\right)$$

(2) $$i_{Csh}\left(v_{sh},v\right)=I_{ph}-i_{D}\left(v_{sh}\right)-\frac{v_{sh}}{R_{sh}}-\frac{v_{sh}-v}{R_{s}}$$

(3) $$\frac{dv_{sh}}{dt}\left(v_{sh},v\right)=\frac{1}{C_{sh}}\left[I_{ph}-i_{D}\left(v_{sh}\right)-\frac{v_{sh}}{R_{sh}}-\frac{v_{sh}-v}{R_{s}}\right]$$

(4) $$I=I_{ph}-I_{o}\left(e^{\frac{{V+IR}_{s}}{v_{T}}}-1\right)-\frac{{V+IR}_{s}}{R_{sh}}$$

### 2.2. Numerical Current Predictor (NCP)

The time series of the panel output voltage V and panel output current I contain N samples. According to [17], for a given model parameter vector P and voltage vector V, the numerical solution of the current vector I for Equations (1)–(4) could be solved by the numerical current predictor (NCP); the implementation steps of the NCP for determining *i_p_*[*k*] are described in Algorithm 1. The model parameters could be evaluated by using the estimation error cost E.
Vvoltage *v*[*k*] time series V =[*v*[0], *v*[1], … *v*[*k*], … *v*[*N*]]I current *i*[*k*] time series I =[*i*[0], *i*[1], … *i*[*k*], … *i*[*N*]]V_sh_
voltage time series *v_sh_*[*k*] across *C_sh_*
V_sh_ =[*v_sh_*[0], *v_sh_*[1], … *v_sh_*[*k*], … *v_sh_*[*N*]]I_p_
predicted panel current *i_p_*[*k*] I_p_ =[*i_p_*[0], *i_p_*[1], … *i_p_*[*k*], … *i_p_*[*N*]]Pmodel parameter vectorP *=*
[I_ph_ I_o_ V_T_ R_sh_ C_sh_ R_s_]Eestimation error costE = 1/*N* (I_p_ − I)(I_p_ − I)^T^ = ∑(I_p_[k] − I[k])^2^/*N*

**Algorithm 1.** Implementation steps for numerical current predictor.Step 1: The current through the capacitor *C_sh_* is assumed to be zero. *v_sh_*[0] is determined by using (1) and (3). Thus,
(5)$$C_{sh}\frac{dv_{sh}}{dt}\left(v_{sh},v\right)=0$$
(6)$$I_{o}\left(e^{\frac{v_{sh}\left[0\right]}{v_{T}}}-1\right)+\frac{v_{sh}\left[0\right]}{R_{sh}}-\frac{v_{sh}\left[0\right]-v\left[0\right]}{R_{s}}=I_{ph}$$
Step 2: *i_p_*[*k*] is calculated by (7)$i_{p}\left[k\right]=\frac{v_{sh}\left[k\right]-v\left[k\right]}{R_{s}}$Step 3: *v_sh_*[*k* + 1] is obtained by solving a trapezoidal equation with Newton’s method (8)$v_{sh}\left[k+1\right]-v_{sh}\left[k\right]=\frac{h}{2}\left[\frac{{dv}_{sh}}{dt}\left(v_{sh}\left[k\right],v\left[k\right]\right)+\frac{{dv}_{sh}}{dt}\left(v_{sh}\left[k+1\right],v\left[k+1\right]\right)\right]$
where *h* is the sampling time interval. The derivative functions on the right-hand side of (8) are obtained by using (3) in the discrete form.

Step 4: *k* is increased by 1.

Step 5: Steps 2 to 4 are repeated until *k* = *N*.

### 2.3. Steady-State Characteristics

For the steady-state characteristics of the PV panel, the junction capacitance is not taken into account and the steady-state output current and voltage equation is shown as follows.

$$I\left(1+\frac{R_{s}}{R_{sh}}\right)=I_{ph}-I_{o}\left(e^{\frac{{V+IR}_{s}}{v_{T}}}-1\right)-\frac{V}{R_{sh}}\;\alpha =\frac{R_{s}}{R_{sh}}$$

(9) $$I\left(1+\alpha \right)=I_{ph}-I_{o}e^{\frac{{V+IR}_{s}}{v_{T}}}-I_{o}-\frac{V}{R_{sh}}\Rightarrow I=\frac{1}{1+\alpha }\left[I_{ph}-I_{o}e^{\frac{{V+IR}_{s}}{v_{T}}}-I_{o}-\frac{V}{R_{sh}}\right]$$

Assume that *R_s_* << *R_sh_* and *α* ≈ 1, when the output voltage is zero, *V* = 0. Then, the current *I* is given by the following:

(10) $$I=I_{sc}=\frac{1}{1+\alpha }\left[I_{ph}-I_{o}e^{\frac{{IR}_{s}}{v_{T}}}-I_{o}\right]{\Rightarrow I}_{SC}<I_{ph}$$

When *I* = 0 (open circuit), the output voltage *V_OC_* is as follows:

$$0=\frac{1}{1+\alpha }\left[I_{ph}-I_{o}e^{\frac{V}{v_{T}}}-I_{o}-\frac{V}{R_{sh}}\right]\Rightarrow 0=I_{ph}-I_{o}e^{\frac{V}{v_{T}}}-I_{o}-\frac{V}{R_{sh}}$$

(11) $$I_{o}e^{\frac{V}{v_{T}}}=I_{ph}-I_{o}-\frac{V}{R_{sh}}{\Rightarrow I}_{o}e^{\frac{V}{v_{T}}}<I_{ph}\Rightarrow V_{OC}<v_{T}ln\left[\frac{I_{ph}}{I_{o}}\right]$$

It can be noted that a typical IV curve consists of two parts. In the first part, the output current remains approximately constant near to *I_ph_*. In the second part, the PV current decreases to 0 within a relative narrow voltage range and the PV current becomes zero at the *V_oc_*. Suppose output current decreases to *βI_ph_* = (1 − *δ*) *I_ph_* (e.g., *δ* = 0.05 = 5%) and the corresponding voltage is as follows:

$$I=\beta I_{ph}=\frac{1}{1+\alpha }\left[I_{ph}-I_{o}e^{\frac{{V+IR}_{s}}{v_{T}}}-I_{o}-\frac{V}{R_{sh}}\right]$$

$$\beta I_{ph}\approx I_{ph}-I_{o}e^{\frac{{V+IR}_{s}}{v_{T}}}-I_{o}-\frac{V}{R_{sh}}$$

(12) $$I_{o}e^{\frac{{V+IR}_{s}}{v_{T}}}+I_{o}+\frac{V}{R_{sh}}\approx {\delta I}_{ph}\Rightarrow e^{\frac{{V+IR}_{s}}{v_{T}}}<\frac{{\delta I}_{ph}}{I_{o}}\Rightarrow V_{x}<v_{T}ln\left[\frac{{\delta I}_{ph}}{I_{o}}\right]$$

For 0 < *V* < *V_x_*, the IV current remains relatively constant near to *I_ph_.* The power *P = IV* is increased with *V*. Therefore, the voltage for maximum power should be in the range *V_x_* < *V_pmax_* < *V_oc_.* The searching process of *V_pmax_* for a standalone PV panel without partial shade should start from *V_oc_* and *V* should be gradually decreased by the step size of Δ*V* and move towards 0.

### 2.4. Proposed System Structure

There are some past research works for PV model parameter estimation that use analytical and conventional methods. In [15], the author introduces a proposed approach to estimate the optimal parameters of photovoltaic (PV) modules using in-field outdoor measurements and manufacturers’ datasheets as well as employing the nonlinear least squares fitting algorithm. The main goal is to determine the optimal parameter values of the implemented model, which are series resistance, reverse saturation current, photocurrent, ideality factor and shunt resistance in the case of the five parameters model. A typical analytical approach requires the entire experimental I–V curve and the information of the slope of the I–V curves of the open circuit and the short circuit points. In [15], the approximate analytical solutions for the parameters of a photovoltaic (PV) five-parameter double-diode model are proposed. These solutions could be used as initial estimates for finding numerical solutions based on the Newton–Raphson method. The proposed formulations are developed based on the information of the open circuit voltage (*V_oc_*), the short circuit current (*I_sc_*), and the current and voltage at the maximum power point (*I_m_*, *V_m_*). However, in order to estimate the dynamic model parameters in Figure 1 with shunt capacitance value, the dynamic current and voltage time series approach is used in this paper.

**Figure 2:**
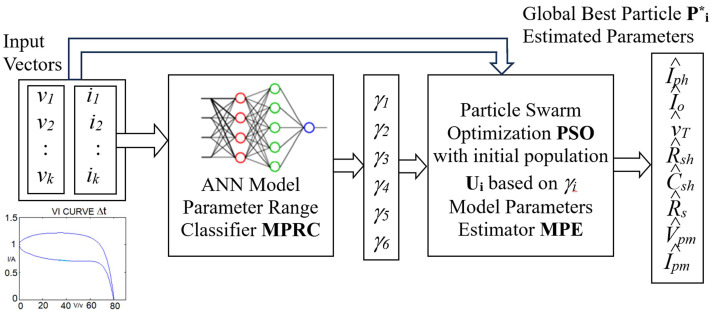
Block diagram of the proposed system.

The proposed system in Figure 2 consists of two stages, the ANN model parameter range classifier (MPRC) and the PSO model parameter estimator (MPE). The inputs to the MPRC are the voltage (V) and current (I) vectors and the output is the range index *γ_i_* of each parameter *γ_i_* ∈ {1, 2, …*N_r_*}, (*γ_i_* − 1)Δp*_i_* ≤ p*_i_* ≤ (*γ_I_* Δp*_i_*) and Δ*p_i_* = (*P_imax_* − *P_imin_*)/*N_r_*. The model parameter estimator is a modified version of the classical PSO. It consists of a modified initialization stage in which the initial population is selected according to the outputs of the MPRC. Based on the outputs of the MPRC, *N_p_* random samples (population size) are selected for the range *γ_i_* to use as initial population *U_i_* for the model parameters *p_i_*. The initial population with *N_p_* particles (or parameter vectors) P is selected. After completing the initialization stage, the PSO algorithm is then used to optimize model parameter vector P to minimize the estimation error. The final global best particle P^*^ of the PSO algorithm is used as the optimal PV model parameter vector.

X = [V I] = [*v*[0], *v*[1], … *v*[*k*], … *v*[*N*], *i*[0], *i*[1], … *i*[*k*], … *i*[*N*]]

(13) $$P=\left[\begin{array}{ccc} p_{1} & p_{2} & \begin{array}{ccc} p_{3} & p_{4} & \begin{array}{cc} p_{5} & p_{6} \end{array} \end{array} \end{array}\right]=\left[\begin{array}{ccc} I_{ph} & I_{o} & \begin{array}{ccc} V_{T} & R_{sh} & \begin{array}{cc} C_{sh} & R_{s} \end{array} \end{array} \end{array}\right]$$

### 2.5. ANN-Based Model Parameter Range Classifier (MPRC)

**Figure 3:**
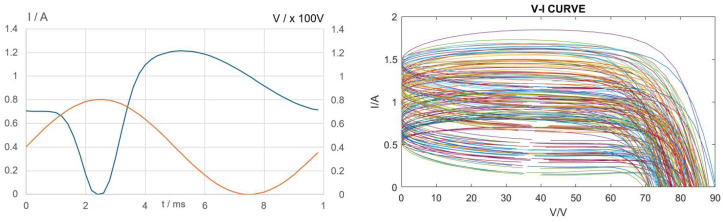
Output current (blue) and voltage (green) curves of the PV panel under load variations.

The design of ANN structures has been one of the most popular research topics in artificial intelligence in the past decade [48]. Past research has been done [1] on the use of ANNs for estimating PV panel model parameters according to variations in temperature and radiation. In this paper, the PV panel model parameters are estimated based on the dynamic I–V variations. Suppose an electronic load with sinusoidal voltage characteristics is applied to the outputs of the PV panel, and the output voltage and current are measured. Due to the junction capacitance and the non-linear diode characteristics, the PV panel output current exhibits non-sinusoidal characteristics due to the junction capacitance and the non-linear diode characteristics as shown in Figure 3. In [49], a new method is proposed to generate a periodic voltage perturbation in two PV panels by connecting suitable switching devices between them. The dynamic I–V data can then be measured with digital acquisition modules installed at the terminal of the PV panels. A new set of approximate analytical solutions for the parameters of the PV five-parameter double-diode model is proposed in [50].

**Figure 4:**
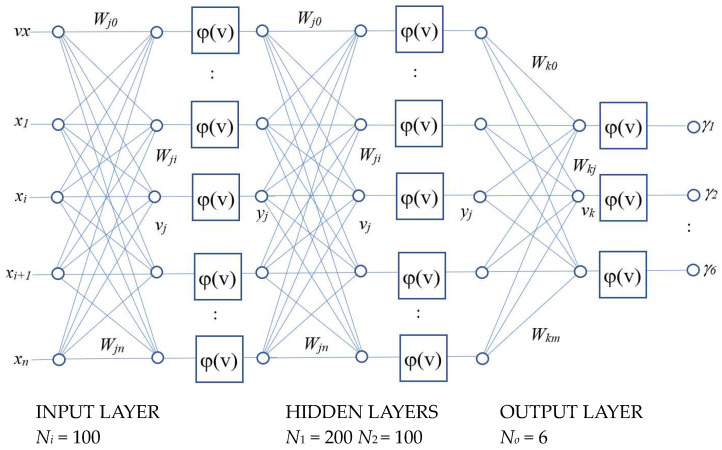
Structure of the model parameter range classifier (MPRC).

The major objective of the MPRC is to determine the range index of the six PV model parameters according to the dynamic I–V vector inputs. In this paper, a three-layer ANN is designed to perform the function of a model parameter range classifier (MPRC) as shown in Figure 4. The MPRC is used to identify the parameter ranges R = φ_2_(P) = [γ_1_ γ_2_ γ_3_ γ_4_ γ_5_ γ_6_] of the PV panel model parameters P = [*I_ph_ I_o_ V_T_ R_sh_ C_sh_ R_s_*], where *γ_i_* ∈ {*1,2…N_r_*} and (*γ_i_* − 1)Δ*p_i_/N_r_* ≤ *p_i_* ≤ *γ_i_* Δ*p_i_/N_r_*, Δ*p_i_ = p_imax_ − p_imin_* and *N_r_* is the number of partitions (5 ≤ *N_r_* ≤ 10) for the parameter range. The set of the parameter range vector R will consist of (N_r_)^6^ patterns, and these patterns indicate the PV model parameter range labels of the I–V curves. The ANN consists of an input layer with 100 nodes for the voltage and current vectors inputs (Input 100, V[0]…V[49], i[0]…i[49]), three hidden layers and output layers with 6 nodes for the range index of the PV panel model parameters. The ANN training data are generated by the simulated models in which the PV panel model parameters are allowed to be varied randomly within the maximum and minimum values. The generated voltage **V** and current **I** time series are used as inputs for ANN training. The parameter range index (γ_i_) of the simulated model parameters γ_i_ = *trunc*{[*p_i_ − p_imin_*]/[*p_i__max_ − p_imin_*]/*N_r_*} is also generated **γ_I_** = [γ_1_ γ_2_ γ_3_ γ_4_ γ_5_ γ_6_], which will be used as target outputs for ANN training. The sinusoidal load voltage amplitude and dc offset are chosen to ensure the variation in output voltage and current will always be positive and below the practical maximum limits (*V_max_* ≥ *V* ≥ 0 and *I_max_* ≥ *I* ≥ 0, 0–3 A, 0–80 V) as shown in Figure 3. The I–V curves are generated for different parameter variations and Figure 4 shows the region of fluctuation for the generated I–V curves. The generated I–V dataset is divided into two parts, with 80% of the dataset used for ANN training while 20% of the dataset is used for testing and performance evaluation. The ANN training algorithm is implemented by using Python (Library used: numpy, pytorch). The outputs **γ_i_** of the ANN will be used to initialize the population for PSO algorithms. The performances of the proposed method will be evaluated and discussed in Section 3.

### 2.6. Design of Model Parameter Estimator by Using Particle Swarm Optimization Method

The particle swarm optimization (PSO) algorithm was developed in 1995 [51] and is one of the most important developments in computational intelligence. This method simulates the social behavior and movement of a flock of birds, which can be used for searching for the global minimum of complex nonlinear functions. PSO has the merit of being simple to implement, but at the same time, there is a tradeoff between global and local searches, and therefore, it does not handle optimization problems with multiple peaks well. As the classical PSO is easily trapped in local optimal points and premature convergence for complex multi-modal non-linear functions, there has been much research on variants or modified PSO methods.

A new method with nonlinear variation in inertia weight along with a particle’s old velocity has been proposed [52] to improve the convergence speed and fine-tune the search in the multi-dimensional space. In [53], time-varying coefficients and inertia weights are proposed for classical PSO. The concept of mutation is proposed, and a small perturbation is added to a randomly selected modulus of the velocity vector of a random particle. The social part and cognitive part of PSO are considered to estimate the new velocity of each particle. Particles are reinitialized when they stagnate in the searching space. In [54], a dynamic multi-swarm PSO is proposed. The whole population is divided into a number of sub-swarms, which are regrouped frequently by various regrouping schedules, and information is allowed to exchange among the particles in the whole swarm and the quasi-newton method is used to improve the local searching ability. The comprehensive learning PSO is proposed in [55], in which a novel learning strategy using all other particles’ historical best information is used to update the particle velocity. This method can enable diversity in the swarm and discourage premature convergence. In [56], the PSO method is augmented with different mutation operators to prevent premature convergence in the searching process.

An approach based on coevolutionary PSO is proposed in [57]. It is used to solve the constrained optimization problem in the form of min–max problems. An adaptive multi-objective PSO is proposed in [58], which incorporate inertia and the acceleration coefficient as control variables with the usual optimization variables and it evolves through the swarming procedure. In [59], a new hybridization of optimization methodologies, PSO with recombination and dynamic linkage discovery, is proposed.

In the proposed PSO-based memetic algorithm [60], both PSO-based searching operators and some special local searching operators are designed to balance the exploration and exploitation abilities. In [61], a novel variant of PSO, orthogonal PSO (OPSO) is proposed to solve intractable large parameter optimization problems. In [62], an adaptive PSO is proposed, the population distribution and particle fitness are evaluated and a real-time evolutionary state estimation is performed to identify one of the four states, including exploration, exploitation, convergence and jumping out, for each generation. This method enables the automatic control of inertia weight, acceleration coefficients and other algorithmic parameters at run time so as to improve the search efficiency and convergence speed. Furthermore, an elitist learning strategy is performed when the evolutionary state is classified as a convergence state. A novel adaptive PSO method has been proposed [63] to solve parameter estimation in nonlinear systems. This method proposed an adaptive mutation mechanism and a dynamic inertia weight to be integrated into the conventional PSO method. In [64], the inertia weight is adaptively adjusted using a fuzzy logic controller during the PSO searching process.

A new PSO algorithm with an adaptive inertia weight is proposed in [65], the proposed method uses feedback on particles’ best positions for controlling the inertia weight. The proposed method also aims at balancing the local search and global search abilities and alternating them throughout the search progress. For the multi-objective PSO, a novel parallel cell coordinate system [66] has been proposed to assess the evolutionary environment including density, rank and diversity indicators based on the measurements of parallel cell distance, potential and distribution entropy, respectively. An efficient hybrid method, PSO with fuzzy logic, is proposed in [67] to solve the data clustering problem. A computational study on an adaptation of PSO, which is designed for parallelization on readily-available heterogeneous parallel computational hardware is given in [68]. An adaptive multi-objective PSO algorithm based on a hybrid framework of the solution distribution entropy and population spacing information is developed in [69] for improving the convergent speed and precision. An adaptive PSO is proposed in [70] that can find the expected quality of different locations and tune its exploration–exploitation dilemma.

An adaptive PSO with Gaussian perturbation and mutation is proposed in [71]. Gaussian perturbation and mutation are incorporated to promote the exploration and exploitation capability. In [72], a PSO with a state-based adaptive velocity limit strategy is proposed, and the velocity limit is adaptively adjusted based on the evolutionary state estimation, in which a high velocity limit is set for a global searching state and a low value of velocity limit is set for a local searching state. The limit handling strategies have been modified to improve the capability of avoiding being trapped in local optima. An adaptive PSO based on a competitive and balanced learning strategy is proposed in [73]. In this method, chaotic inertia weight and time-varying acceleration factors are leveraged in the exploration state to speed up the convergence process. The competitive and balanced learning mechanism is exploited to update the velocity of particles. Competition and the average mechanism can alleviate situations in which PSO is trapped in local optima in the late stage of evolution. Next, an adaptive location renewal mechanism is applied to trade off exploration and exploitation. Finally, the proposed method is compared with five competitive PSOs.

**Figure 5:**
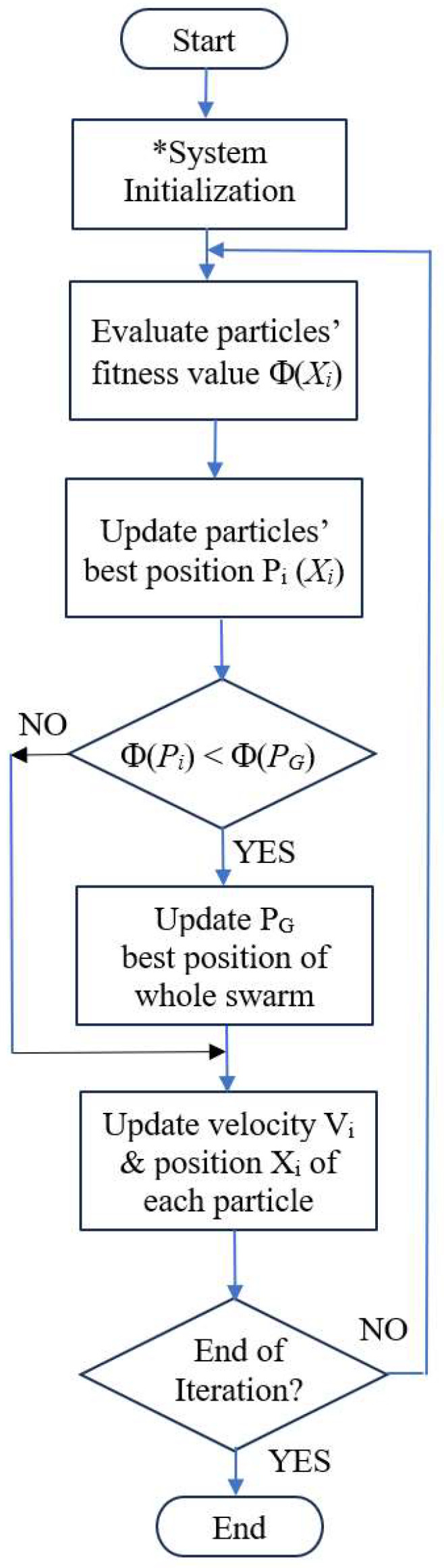
Flow chart of particle swarm optimization. * Note: initialization is based on the outputs of MPRC.

In this paper, as the initial population of particles is selected according to the pre-tuning results of the MPVC, the initial population should be near to the final optimal solutions as compared to the random initialization approach. The PSO algorithm in Figure 5 is used to estimate the PV panel model parameters. It has been found that a classical PSO with random particle initialization for the PV panel model parameters estimation requires a larger number of iterations (10 × 10^3^) [47] to reach the steady state and the overall process requires significant computing resources. On the other hand, the proposed model parameters estimator (MPE) includes a PSO algorithm with a modified initialization stage. It will be shown that using the MPRC for pre-tuning can speed up the convergence significantly. For the ith particle of the PSO method, **X_i_** = [p_i1_ p_i2_ p_i3_ p_i4_ p_i5_ p_i6_] = [I_ph_ I_o_ V_T_ R_sh_ C_sh_ R_s_], the mean square error (MSE) is used as a PSO fitness function (objective value) for the feature values’ selection process:

(14) $$\Phi_{xi}=\frac{1}{N}\sum_{j=1}^{N}{e_{j}}^{2}=\frac{1}{N}\sum_{j=1}^{N}{\left[I_{pj}-I_{j}\right]}^{2}$$

where *N* is the total number of testing data samples for the current series, where *e_j_* is the error between the exact *I*_j_ and estimated (or predicted) current *I_pj_* values. Based on the input voltage vector, the current series are predicted by the estimated model parameters [17], and the fitness values for the particles with estimated model parameters are determined based on Equation (14). The position (*X_i_*) and velocity (*V_i_*) of *i*th particle are updated by the following equations. The step-by-step procedures for the PSO method are shown in Figure 5 and summarized as follows.

*X_i_* = [*X_i_*_1_, *X_i_*_2_, …, *X_ik_*, …, *X_in_*], (15)

*V_i_* = [*V_i_*_1_, *V_i_*_2_, …, *X_ik_*, …, *V_in_*] (16)

*V_ik_*(*t +* 1) *= ωV_ik_*(*t*) *+ C*_1_
*r*_1*k*_ (*P_ik_ − X_ik_*) *+ C*_2_
*r*_2*k*_ (*P_gk_ − X_ik_*) (17)

*X_ik_*(*t +* 1) = *X_ik_*(*t*) *+ V_ik_*(*t +* 1), *i* = 1*…N_p_* (18)

where 

*P_i_* and *Φ_ibest_* are its own best experience, position and objective value;

*P_g_* and *Φ_gbest_* are the best experiences of the whole swarm, position and objective value;

*ω* is the inertia weight; *C*_1_ and *C*_2_ are cognitive and social acceleration coefficients;

*r*_1*d*_ and *r*_2*d*_ are random numbers in [0, 1]; and *N_p_* is the population size.

The first term of Equation (17) is the inertia, which is affected by previous velocity. The second term of Equation (17) is the local acceleration factor, which is affected by the particle’s best position, while the last term is affected by the global acceleration factor, the effect of the globally best particle with the best function evaluation.

1. Initialize the particles’ velocities *V_i_* and positions *X_i_.*
2. Update particles’ *V_i_* velocities and positions *X_i_* by Equations (17) and (18).
3. Compare the estimated current (${\hat{I}}_{j}$) with the actual current (*I_j_*) for all of the model parameter vectors (*j* = 1…*N*). Compute the objective values *Φ_Xi_* for each particle *X_i_* by Equation (14).
4. Update the best position *P_i_* and the best objective value *Φ_ibest_* for each particle *X_i_*.
5. Update the global best position *P_g_* and the global best objective value *Φ_gbest_.*
6. Go back to step 2 to 5 for updating cycle until maximum generation is reached.

### 2.7. Proposed PV Panel Model Parameters Estimation Method

The step-by-step procedures of the proposed PV panel model parameters estimation algorithms (ANN–PSO) are as follows.

1. Apply a sinusoidal load perturbation to the output of the PV panel, measure the voltage and current time series. The current i[k] time series and voltage v[k] time series will be sampled by the current and voltage sensors. The current and voltage X = [I V] vectors will be formed, where

V = [v[0], v[1], … v[k], … v[N]]    I = [i[0], i[1], … i[k], … i[N]]

**Figure 6:**
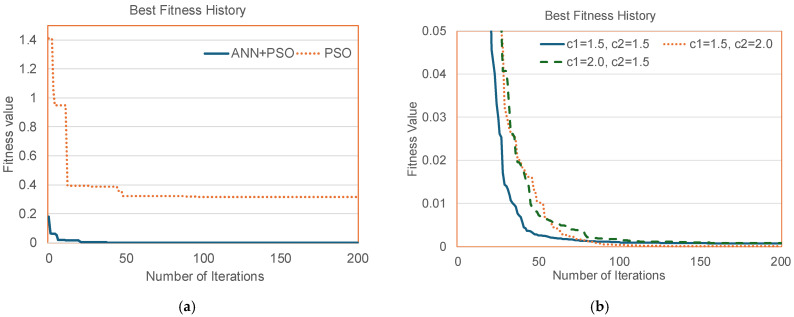

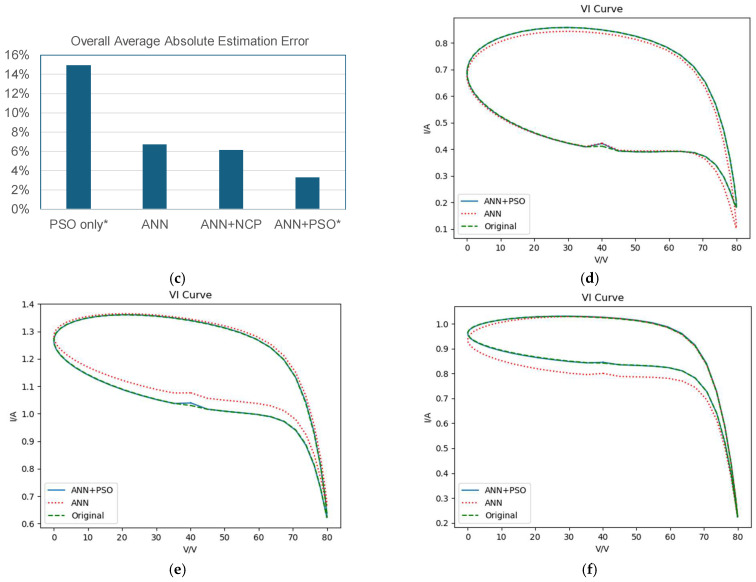
Samples of original (calculated by exact model, hidden) and estimated (ANN: dotted red, ANN+PSO: solid blue) *VI* curves. (**a**) Best fitness value history for Sample 1; (**b**) average of the best fitness values for 20 samples (ω = 0.5) with different C_1_ and C_2_ parameters; (**c**) distribution of the overall average absolute estimation errors for different methods; (**d**) sample 1; (**e**) sample 2; and (**f**) sample 3.

2. Use MPRC with I–V vector as input to estimate the model parameters’ range vector [γ_1_ γ_2_ γ_3_ γ_4_ γ_5_ γ_6_].
3. Use the MPE to estimate the PV panel model parameters. According to the model parameters range γ_i_, initialize *N_p_* particles for the PSO algorithms (Figure 6), which is used to estimate the model parameters vector P* of the PV panel.
4. Using MSE as the fitness function for the PSO, *J* = ∑ [I_p_ − I*]^2^/*N* where I_p_ = current predicted by the model and I^*^ = actual measured current. Update the position (X_i_ = [I_ph_ I_o_ V_T_ R_sh_ C_sh_ R_s_]) and velocity (V_i_) of the ith particle by the following equations.

X_i_ = [*X_i_*_1_, *X_i_*_2_, *…X_ik_…X_in_*]     V_i_ = [*V_i_*_1_, *V_i_*_2_, *…X_ik_…V_in_*]

*V_ik_*(*t* + 1) = *ωV_ik_*(*t*) + *C*_1_*r*_1*k*_(*P_ik_* − *X_ik_*)*+C*_2_*r*_2*k*_(*P_gk_* − *X_ik_*)   *X_ik_*(*t +* 1) *= X_ik_*(*t*) *+ V_ik_*(*t +* 1)

*i* = 1…*N_p_*, *P_i_* and *P_best_* are its own best experience, position and objective value;

*P_g_* and *g_best_* are the best experiences of the whole swarm, position and objective value;

*ω* is the inertia weight; *C*_1_ and *C*_2_ are cognitive and social acceleration coefficients,

and *r*_1*d*_ and *r*_2*d*_ are random numbers in [0, 1]. The PSO iterations repeat until the maximum number of generations is reached.

5. The global best particles vector X* will be used as the optimal PV panel model parameters vector P*. Furthermore, the voltage for maximum power operation *V_p_* could be estimated by using P*.

## 3. Results and Analysis

For the evaluation of the algorithm proposed in this paper, the ANN–PSO algorithm was implemented on a server with 2.6 GHz Intel CPU i7-8700 (Intel, Santa Clara, CA, USA) and 32 GB memory. The ANN–PSO algorithm was developed based on the Python libraries (Library used: numpy, pytorch). The I–V dataset was generated by PV panel model simulations and the current predictor in [17]. In [17], the ANN was designed to approximate the mapping between IV data patterns and the PV model parameters P. The estimated PV model parameters P are then passed to the numerical current predictor (NCP). The NCP is designed to fine-tune the PV model parameters P estimated by the ANN to reduce the prediction current error. In order to evaluate the performances of the proposed ANN–PSO, the ANN–PSO was compared with the single ANN approach and the ANN–NCP algorithms in [17]. For the single ANN approach, a traditional single-stage ANN was trained to approximate the non-linear mapping between input *I–V* parameters and the estimated model parameters *P*.

The PV model parameters *I_ph_ =* 1, *I_o_* = 1 × 10^−7^, *V_T_* = 5, *R_sh_* = 1000, *C_sh_* = 1 × 10^−6^ and *R_s_* = 1 were given ±90% variations while *v_T_* was given a ±5% variation for generating the dataset. The dataset consisted of 2748 data lines with each data line consisting of 50 (*N*) voltage samples (*V*[0]…*V*[49]) and 50 currents samples (*I*[0]…*I*[49]), of which 2199 (80%) data lines were used for training and 549 (20%) data lines were used for testing and performance evaluation. The ANN settings of the ANN–PSO approach and single ANN approach were the same. The ANNs had an input dimension of one hundred (*V*[0]…*V*[49] *I*[0]…*I*[49]), two hidden layers, a first hidden layer with two hundred nodes and a second hidden layer with one hundred nodes, and an output layer with six nodes. The learning rate of the ANNs was 0.01. For MPRC, the system parameters were as follows: *N_r_* = 10, *g_i_* ∈ {1, 2, 3…10}. For the PSO algorithms, the following settings were used: swarm size = *N_p_* = 20, maximum number of iterations = *N_max_* = 200, PSO cognitive parameters *C*_1_ = 1.5, social parameter *C*_2_ = 1.5 and inertia parameter *w* = 0.5.

The simulation results of the estimation error of the model parameters for 20 data samples are summarized in Table 1. The estimated PV panel model parameters are listed in the left-hand columns, the estimation errors of each parameter are listed in the right-hand columns. The average absolute estimation errors (*Avg*|*E*(*P*)|) are the average of the absolute values of the estimation error of each parameter in the left-hand columns. The average absolute estimation error is given by the following:

(19) $$Avg\left|E\left(P\right)\right|=\frac{1}{6}\sum_{j=1}^{6}\left|\frac{p_{i}-p_{i}^{*}}{p_{i}^{*}}\right|$$

where *p_i_* are the estimated values and p^*^_i_ are the exact values.

In Table 2, the overall average absolute estimation errors are the overall averages of the $\bar{E}\left(P\right)$ for the whole testing dataset (*N* = 549 testing data samples). The overall average of the absolute estimation error of the ANN–PSO is compared with that of single PSO, direct ANN and ANN–NCP methods. According to Table 2, it can be noted that the direct ANN method has an overall average estimation error of 6.7%; while the ANN-–NCP gives an overall average estimation error of 6.15%, the ANN–PSO gives an overall average estimation error of 3.3% and the classical PSO gives 14.9% with 200 iterations for a ±90% variation range. It can be noted that the ANN–PSO method gives a better accuracy as compared to the direct ANN method, ANN–NCP method and classical PSO method with a maximum number of iterations at 200. The distributions of the overall average of the absolute parameter estimation errors for different methods are shown in Figure 6.

A sample of the best fitness values vs. number of iterations is shown in Figure 6. It can be noted that the best fitness (ISE) value starts with 1.4 for the classical PSO and 0.2 for the proposed ANN–PSO method. Furthermore, the proposed method comes to a steady state with a best fitness value less than 1.4 × 10^−4^ in about 100 generations while the classical PSO approach requires a significantly larger number of iterations for convergence. At an iteration of 200, the ANN–PSO gives a best fitness value of 1.3 × 10^−4^ while that of the PSO method gives a best fitness value of 0.316. The maximum number of iterations is set at 200 in this paper. If the PSO is allowed to run beyond 200 iterations, the best PSO fitness decreases to 0.097 while the best ANN–PSO fitness reaches 1.29 × 10^−4^ at 500 iterations. The proposed ANN–PSO method has a better performance than the direct ANN and classical PSO approaches. Samples of I–V curves for the proposed ANN–PSO method and the direct ANN method are shown in Figure 6. According to the simulation results in Figure 6, it can be noted that the ANN–PSO method closely matches the original exact I–V curves while the direct ANN method gives I–V curves with some deviations from the original exact I–V curves.

A fault diagnosis of PV Panels using modified PSO and dynamic I–V characteristics has been proposed in [47], which can provide estimated model parameters for the PV panel health diagnosis. In [47], a mutation method is applied to the global best particles with different step sizes of particle perturbations. The fitness values of these mutated particles will be compared with the current global best particle. The current global best particle will be replaced by the mutated particle if the mutated particle can give even better fitness value. The inertia weight factor is determined according to the method in [62]. In [47], the experimental results with varying electronic loads show that the levels of deviation in PV panel parameters can indicate healthy or faulty conditions of the PV panels. The algorithm in [47] involves the application of the modified PSO method which requires the calculations of the predicted current vectors and the objective function values for all chromosomes of the population for a given number of generations. As indicated in [47], the modified PSO approach needs a significant number of iterations (>500) before estimation error can approach a satisfactory level, and therefore, the PSO approach requires significant computational load. To evaluate the performance of the proposed ANN–PSO method, the experimental dataset in [47] was used to test the performance of the proposed method.

**Figure 7:**
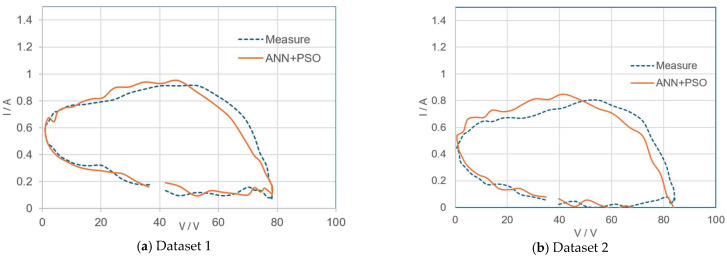
Measured VI curve with reference experimental data from [47] and VI curve estimated by proposed method.

The experimental dataset in [47] is composed of voltage and current data measured under a load perturbation test for practical PV panels (Sungen SG-NH80-GG 80 W, a-Si type). In the experiment, the panels were connected to an electronic load HP6050A controlled by a computer. Experimental I–V data in [47] were inputted to the ANN–PSO system and the estimation results are summarized in Table 3 and Figure 7. It can be noted that the model estimate using the proposed method gives a smaller RMSE (root mean square error) of predicted current than that of [47], but the proposed method requires a smaller number of iterations (N_max_ = 200) to converge. The RMSE of the proposed method is about 0.055 (dataset 1) while that of the model predicted by [47] is about 0.21 (dataset 1).

## 4. Discussion

According to the results of the previous section, the performance of the proposed ANN–PSO system has better estimation accuracies than the original ANN approach and ANN–NCP approach. According to the results in Table 2, ANN–PSO can give better accuracies with a relatively small number of iterations. The ANN–PSO gives better accuracies than the single PSO approach for a relatively small number of iterations (*N_max_* = 200). Furthermore, the convergence speed of ANN–PSO is faster than a classical PSO.

Most of the variants or modified PSO algorithms in the past research aimed at preventing premature convergence and being trapped in local optima. This situation may occur for the conventional PSO. On the other hand, with the pre-tuning actions of the MPRC, the particles of the initial population are reset at positions near to the optimal solutions. According to the simulation studies in Section 3, it can be noted that the initial best fitness values of ANN–PSO are much smaller than those of the conventional PSO approach. Simulation studies show that the classical PSO method incorporating the ANN-based MPRC is good enough for fine-tuning and searching for the optimal solutions of the PV model parameters. Furthermore, the proposed scheme can come to steady state with a similar accuracy level as a PSO with a smaller number of iterations.

From the results in Figure 6, the convergence speed of the best fitness of the proposed method is faster than the classical PSO approach. The proposed ANN–PSO requires a smaller number of iterations for convergence, and therefore, the proposed method needs less computational resources as compared to the single PSO approach. The curves of the average best fitness values for 20 samples vs. the number of iterations for ANN–PSO and classical PSO methods are shown in Figure 6. It can be noted that at 200 iterations, ANN–PSO gives the average best fitness of 3.45 × 10^−4^ and PSO gives the average best fitness of 8.39 × 10^−4^. If the algorithms are allowed to run beyond 200 iterations, it has been found that ANN–PSO gives the average best fitness of 1.53 × 10^−4^ and PSO gives the average best fitness of 6.98 × 10^−4^. According to the results in Table 2, the ANN–PSO gives a more accurate PV panel model as compared to the model estimated by a pure ANN approach. The results in Figure 6 show that the IV curves obtained by the ANN–PSO method closely matched with the IV curves of the exact model, while the IV curves of the pure ANN approach have some deviation from the IV curves of the exact model.

The PSO parameters are selected based on the best fitness evaluations and past research experiences in [47]. According to the results in Table 4, the PSO parameters are selected based on the studies on the variations in the best fitness values with different combinations of C_1_, C_2_ and ω as shown in Table 4. In Table 4, the parameters C_1_ and C_2_ are varied in the range of 1.0 ≤ C_1_ ≤ 2.0 and 1.0 ≤ C_2_ ≤ 2.0. The variations in the best fitness values of sample 1 for different combinations of C_1_ and C_2_ are summarized in Table 4. For a fixed setting of c_1_ = c_2_ = 1.5, the variations in the best fitness of sample 1 for different values of inertia ω (0.2 ≤ ω ≤ 0.8) are summarized in Table 4b. Extensive simulations show that the parameter settings ω = 0.5, 1.5 ≤ C_1_ ≤ 2 and 1.5 ≤ C_2_ ≤ 2 give the best performance. In this paper, the settings ω = 0.5, C_1_ = 1.5 and C_2_ = 1.5 were used. According to Figure 6, the settings of C_1_ = C_2_ = 1.5 give the fastest convergence of best fitness. It has been found that the convergence speed and transient behavior of the best fitness is affected by the selection of PSO parameters. However, if the PSO is allowed to run for a large number of iterations (e.g., 10^4^), the steady-state best fitness for the proposed parameter ranges tends to converge to similar accuracy levels.

For the selection of ANN parameters, results for the simulation analysis are shown in Table 4. The loss values are evaluated for different settings of ANN parameters. In Table 4b, different settings for the number of nodes in the first, second and third layers are considered, the learning rate is kept at 0.01 and the loss values for ANN training are evaluated. It has been found that two hidden layers with 200 nodes in the first hidden layer and 100 nodes in the second hidden layer are appropriate choices of ANN structure. Having selected the number of hidden layers and number of nodes in the hidden layers, the learning rate is varied from 0.001 to 0.10, and the loss values for ANN training are evaluated. Simulation results show that a learning rate of 0.01 is an appropriate choice for ANN training. Simulations studies also show that the settings No. of hidden layers = 2, No. of nodes in first hidden layer = 200, No. of nodes in the second hidden layer, and learning rate = 0.01, give optimal performance for the ANN operation. These settings are used for the ANN–PSO and single ANN approaches.

According to the results in [47], *v_T_* will remain approximately constant for different cases of health conditions for PV panels. Therefore, combinations of the values of *C_sh_* and *R_s_* could be used for diagnosing PV panel health (e.g., large *R_s_* and smaller *C_sh_* for an unhealthy PV panel, a PV panel with physical damage will change the value of R_s_). Therefore, measuring the I–V responses after load perturbations could provide data for ANN–PSO algorithms, and from this data, the PV panel parameters could be obtained. Having obtained the model parameters, the voltage for maximum power operations could be derived by tracking the MPPT voltage point on the PV output current formula. Therefore, the duty cycle for the MPPT operation of the converter connecting the PV panel and output load could be derived and the overall method could be used for an MPPT control and fault diagnosis system.

## 5. Conclusions

This paper proposed an ANN–PSO method for PV panel parameters estimation. This method can be applied to PV panel health monitoring and MPPT control. According to the results in Section 3, the proposed method has better estimation accuracies as compared to the single ANN approach and the ANN–NCP approach in [17]. A numerical layer for PV modelling is proposed in [17] and the parameter estimation algorithm for ANN–NCP has been investigated in [17]. The tuning of the numerical layer is based on the greatest descent local searching approach and the ANN–NCP has better accuracy than the direct ANN method. In this paper, a 10% sub-division (*N_r_* = 10) is used for MPRC output. The major function of the model parameter range classifier (MPRC) is to estimate the approximate solutions of the PV panel model parameters. The outputs of MPRC are the parameter range indices and these output data will be used for the next stage involving the model parameter estimator (MPE). In this paper, the MPE is a PSO parameter estimator with a modified initialization stage. The initial particle populations are selected according to the output of the MPRC so that the initial population of the particles will be near to the optimal solution. According to the simulation studies and data analysis based on the experimental dataset, the initial estimation error for the best particle of the proposed method is much smaller than that of the classical PSO approach. Since the positions of the initial particles’ populations are near to the optimal solution, the PSO search process needs a smaller number of iterations for convergence. Furthermore, the proposed ANN–PSO approach can give better accuracies than the classical PSO approach with a smaller number of iterations. As the proposed method can give good accuracies with a faster convergence speed, it requires less computational load and complexity as compared to the classical PSO approach. In this paper, the algorithms are evaluated based on the considerations of accuracy, convergence speed and computational load. A relatively small population size of 20 is selected in this paper, as the pre-tuning action of MPRC can give an approximate optimal solution for the parameter estimation problem. A classical PSO method with a relatively small population size is sufficient for the fine-tuning of estimated parameters. This feature also reduces the computational load and complexity indirectly.

For the selection of PSO parameters, the best fitness values for the testing dataset were evaluated for different combinations of PSO parameters. For the selection of ANN structure and hyper-parameters, data analysis on the variations in the loss values for different settings of ANN training parameters was carried out. With appropriate ANN and PSO settings, simulation results show that the proposed ANN–PSO method (1.3 × 10^−4^) can give a better fitness than that of the classical PSO approach (0.316) with 200 iterations. The average absolute estimation error of ANN–PSO is 3.3% while those of the ANN–NCP and ANN approaches are 6.1% and 6.7%, respectively. For the data analysis based on the experimental dataset, the proposed method can give an RMSE of 0.055 for the predicted current while that of [47] gives an RMSE of 0.21 with 200 iterations. On the other hand, the single PSO approach needs more than 500 iterations for convergence.

The proposed method is based on the analysis of the current and voltage time series after load perturbation, the model parameters are estimated based on numerical models, the ANN classifier and PSO algorithms. Online monitoring of the health conditions of solar PV panels is very crucial for the effective operation of solar PV array systems. Real-time current and voltage data can be collected through sensors and IV data will be sent back to the host computer for model parameters estimation. The estimated model parameters obtained by the proposed system can be used to evaluate the health condition and tracking of the MPPT voltage of the PV panel. The estimated parameters are then used for the controller design for optimal performance at MPP. An ANN–PSO PV model parameter estimation algorithm is proposed in this paper. The proposed method can be used for model parameter estimation, output prediction and the health monitoring of solar PV panels. Future research can be conducted for the maximum power point (MMPT) control of PV array systems.

## Figures and Tables

**Table 1:** Estimation error of the model parameters for 20 data samples (ANN + PSO). ANN–PSO (Input 100, V[0]…V[49], i[0]…i[49], two hidden layers, first hidden layer 200 nodes, second hidden layer 100 nodes, output six model parameters’ values, learning rate 0.01, N = 2748, training data 2199, testing data 549.

Model Parameter Values						Parameter Error (%)						Avg|E(P)|
*I_ph_*	*Io*	*V_T_*	*R_sh_*	*C_sh_*	*R_s_*	*I_ph_*	*I_o_*	*V_T_*	*R_sh_*	*C_sh_*	*R_s_*	*Error%*
0.687327	4.43 × 10^−8^	5.028819	647.7544	8.98 × 10^−7^	1.141352	−0.01%	−1.76%	−0.11%	0.08%	0.01%	1.02%	0.50%
1.470966	1.29 × 10^−7^	4.955088	1353.875	1.42 × 10^−6^	1.541955	−0.01%	10.46%	0.61%	1.76%	−0.05%	−1.56%	2.41%
1.097623	1.07 × 10^−7^	5.021067	1144.587	5.63 × 10^−7^	1.056793	0.02%	−2.33%	−0.15%	−0.72%	−0.05%	−0.18%	0.57%
1.450665	1.28 × 10^−7^	5.038308	1653.205	1.38 × 10^−6^	1.691926	0.01%	−11.33%	−0.73%	−2.43%	0.05%	1.89%	2.74%
0.621455	7.25 × 10^−8^	4.955404	1539.586	7.74 × 10^−7^	1.398938	0.01%	−1.11%	−0.07%	−0.30%	−0.01%	0.14%	0.27%
0.593933	9.67 × 10^−8^	4.983181	1671.098	1.68 × 10^−6^	1.440388	0.35%	7.23%	0.48%	−8.00%	−0.12%	−5.70%	3.65%
0.554694	1.55 × 10^−7^	4.986679	1319.29	6.58 × 10^−7^	1.148153	0.03%	−10.99%	−0.76%	−1.59%	0.07%	3.43%	2.81%
1.169152	1.24 × 10^−7^	5.019874	671.1248	1.26 × 10^−6^	1.173266	−0.01%	7.39%	0.45%	0.51%	−0.03%	−1.63%	1.67%
0.813365	1.19 × 10^−7^	5.032863	559.3855	6.04 × 10^−7^	1.141564	−0.01%	2.79%	0.18%	0.14%	−0.02%	−1.00%	0.69%
0.542401	1.18 × 10^−7^	5.024342	1493.297	1.38 × 10^−6^	1.674077	0.02%	−9.47%	−0.63%	−1.68%	0.04%	1.79%	2.27%
0.494695	7.93 × 10^−8^	4.958878	1649.702	1.15 × 10^−6^	0.996171	0.51%	−8.61%	−0.50%	−13.29%	−0.05%	−5.02%	4.66%
0.346326	1.66 × 10^−7^	5.030319	405.0068	1.42 × 10^−6^	1.227686	0.05%	−16.85%	−1.22%	−0.74%	0.11%	4.27%	3.87%
1.076312	5.62 × 10^−8^	4.98256	1443.875	1.10 × 10^−6^	1.027417	−0.01%	−1.88%	−0.10%	0.42%	0.01%	2.36%	0.80%
0.696398	9.02 × 10^−8^	4.99645	1199.748	1.11 × 10^−6^	0.527183	0.00%	−7.20%	−0.47%	−0.58%	0.03%	3.90%	2.03%

**Table 2:** Summary of average estimation absolute error of the model parameters for the testing dataset (793 samples). * Note: number of PSO iterations, *N_max_* = 200; swarm size = *N_p_* = 20; and number of data in input vector, *N* = 100.

	Average Absolute Parameter Error (%)						Overall
	*I* *_ph_*	*I* *_o_*	*V* *_T_*	*R* *_sh_*	*C* *_sh_*	*R* *_s_*	*Avg*|*E(P)*|*%*
Input Range	±90%	±90%	±5%	±90%	±90%	±90%	
PSO only *	0.574%	50.575%	2.565%	17.847%	0.740%	17.343%	14.941%
ANN	5.577%	8.039%	0.482%	9.687%	6.312%	10.131%	6.705%
ANN + NCP	5.337%	11.202%	0.477%	6.822%	5.936%	7.101%	6.146%
ANN + PSO *	0.529%	10.051%	0.599%	3.221%	0.364%	5.039%	3.301%

**Table 3:** Results for the model parameters from measured data: testing of proposed method using experimental data from [47]. Summary of reference parameters in [47] and model parameters estimated by proposed method. Comparisons of root mean square error of predicted current data (RMSE) between estimated model parameters in [47] and the model parameters estimated by proposed method with experimental data in [47] used as input. High temperature condition—the lights are turned on for 2 min and the panels are heated up. (Dataset 1 Sample 1 with cracks.) (Dataset 2 Sample 2 with cracks.)

Panel	Predicted Parameter Values MPSO [51]						*I_est_* *RMSE*	Predicted Parameter Values ANN + PSO						*I_est_* *RMSE*
	*I_ph_*	*I_o_*	*V_T_*	*R_sh_*	*C_sh_*	*R_s_*		*I_ph_*	*I_o_*	*V_T_*	*R_sh_*	*C_sh_*	*R_s_*	
Dataset 1	0.602	7.09 × 10^−8^	5.26	369	2.60 × 10^−7^	2.60	0.2100	0.543	5.893 × 10^−4^	12.210	18752.5	1.470 × 10^−6^	8.012	0.0555
Dataset 2	0.454	1.80 × 10^−8^	5.23	587	4.82 × 10^−7^	4.82	0.2227	0.417	5.573 × 10^−4^	12.462	14125.4	1.450 × 10^−6^	13.272	0.1082

**Table 4:** (**a**) Best fitness values for sample 1 with different combinations of the PSO parameters. (**b**) Simulation results for ANN parameters. Input 100, V[0]…V[49], i[0]…i[49], two hidden layers, first hidden layer two hundred nodes, second hidden layer one hundred nodes, output layer six nodes, learning rate 0.01, total data 2748, training data 2199, testing data 549, and maximum epochs for training 5000.

(**a**)							
***C*_1_**	**1.0**	**1.5**		**2.0**		**ω**	
***C*_2_**							
1.0	54.12 × 10^−4^	126.83 × 10^−4^		1.67 × 10^−4^	0.2	0.5	0.8
1.5	1.94 × 10^−4^	0.597 × 10^−4^		0.12 × 10^−4^	1.09 × 10^−3^	0.0597 × 10^−3^	2.82 × 10^−3^
2.0	100.7 × 10^−4^	0.084 × 10^−4^		8.52 × 10^−4^	C_1_ = 1.5, C_2_ = 1.5, N_max_ = 200, N_p_ = 20		
Ω = 0.5, N_max_ = 200, N_p_ = 20							
(**b**)							
**No. of Hidden Layers**	**No. of Nodes in 1st Hidden Layer**		**No. of Nodes in 2nd Hidden Layer**		**No. of Nodes in 3rd Hidden Layer**	**Learning Rate**	**Loss**
2	200		100		-	0.01	0.7993
2	200		200		-	0.01	0.8387
2	200		150		-	0.01	0.8400
2	150		50		-	0.01	0.8535
2	300		200		-	0.01	0.8989
3	200		100		50	0.01	0.8173
3	200		150		50	0.01	0.8385
3	300		200		100	0.01	0.8101
**Training** (Dropout = 0.1)			**Dropout** (Learning rate = 0.01)				
Learning rate	Loss		Dropout		Loss		
0.100	0.8303		0.10		0.7993		
0.050	0.7842		0.20		0.8431		
0.020	0.8293		0.05		0.8648		
0.010	0.7993						
0.005	0.8613						
0.001	0.8067						

## Data Availability

The datasets presented in this article are not readily available because the data are restricted to the project study only.
